# Relative Distribution Entropy Loss Function in CNN Image Retrieval

**DOI:** 10.3390/e22030321

**Published:** 2020-03-11

**Authors:** Pingping Liu, Lida Shi, Zhuang Miao, Baixin Jin, Qiuzhan Zhou

**Affiliations:** 1 College of Computer Science and Technology, Jilin University, Changchun 130012, China; miaoz16@mails.jlu.edu.cn (Z.M.); jinbx18@mails.jlu.edu.cn (B.J.); 2 Key Laboratory of Symbolic Computation and Knowledge Engineering of Ministry of Education, Jilin University, Changchun 130012, China; 3 School of Mechanical Science and Engineering, Jilin University, Changchun 130025, China; 4 College of Software, Jilin University, Changchun 130012, China; shild18@mails.jlu.edu.cn; 5 College of Communication Engineering, Jilin University, Changchun 130012, China; zhouqz@jlu.edu.cn

**Keywords:** image retrieval, relative entropy, deep metric learning, Euclidean distance

## Abstract

Convolutional neural networks (CNN) is the most mainstream solution in the field of image retrieval. Deep metric learning is introduced into the field of image retrieval, focusing on the construction of pair-based loss function. However, most pair-based loss functions of metric learning merely take common vector similarity (such as Euclidean distance) of the final image descriptors into consideration, while neglecting other distribution characters of these descriptors. In this work, we propose relative distribution entropy (RDE) to describe the internal distribution attributes of image descriptors. We combine relative distribution entropy with the Euclidean distance to obtain the relative distribution entropy weighted distance (RDE-distance). Moreover, the RDE-distance is fused with the contrastive loss and triplet loss to build the relative distributed entropy loss functions. The experimental results demonstrate that our method attains the state-of-the-art performance on most image retrieval benchmarks.

## 1. Introduction

**Figure 1:**
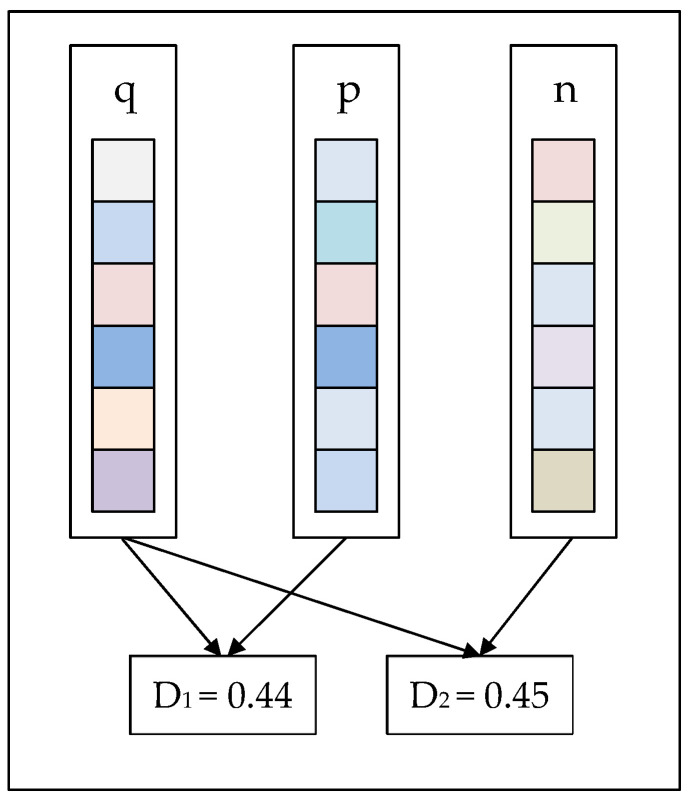
Each rectangle represents a feature descriptor obtained after the convolution of the neural network. Different colors of small squares represent different feature intensities in the descriptor. The Euclidean distance ($D_{1}$) between descriptor n and descriptor q is 0.45, while the Euclidean distance ($D_{2}$) between descriptor p and descriptor q is 0.44. D_1_ is approximately equal to D_2_, but the internal spatial distribution of p and n is obviously different.

In recent years, the newly proposed image retrieval algorithms based on convolutional neural networks [1,2,3,4,5] (CNN) have greatly improved the accuracy and efficiency. This has been the mainstream direction of academic research of image retrieval. In the beginning, CNN could only be applied to image classification tasks [6,7,8]. However, image classification is different from the image retrieval. Krizhevsky [9] flexibly applied a convolutional neural network to image retrieval. AlexNet [9] is designed for image classification and retrieval. Subsequently, Noh [10] proposed a Local Feature descriptor, called DELF (Deep Local Feature), which is suitable for large-scale image retrieval. A large number of studies have illustrated that the output features of the convolutional layer of the neural network have excellent discrimination and scalability. More recently, image retrieval algorithms based on convolutional neural networks emerged one after another. These methods are mainly summarized into three categories: fine-tuned networks, pre-trained networks, and hybrid networks. Among them, hybrid networks are less efficient in image retrieval tasks, and pre-trained networks are widely used. The fine-tuned network initializes the network architecture through the pre-trained image classification model and then adjusts the parameters according to different retrieval tasks. The fine-tuned network usually optimizes the network parameters by training the network architecture of metric learning. Metric learning aims to learn an embedding space, where the embedded vectors of positive samples are encouraged to be closer, while negative samples are pushed apart from each other [11,12,13]. Recently, a lot of deep metric learning methods have been based on pairs of samples such as contrastive loss [14], triplet loss [15], quadruplet loss [16], lifted structured loss [17], N-pairs loss [18], binomial deviance loss [19], histogram loss [20], angular loss [21], distance weighted margin-based loss [22], and hierarchical triplet loss (HTL) [23]. Most of the above-mentioned loss functions take common vector similarity (such as Euclidean distance) as the final image descriptor into consideration. However, it is not accurate enough to measure the similarity between features only by Euclidean distance, which lacks the difference in the internal spatial distribution [24] of the image pair. As illustrated in Figure 1, each rectangle represents a feature descriptor obtained after the convolution of the neural network. The value of Euclidean distance between descriptors for different images may be the equal or small, but the spatial distribution of every descriptor may be greatly different. In the information processing field, entropy [25] is an effective measurement to reflect the distribution information. Relative entropy [26,27,28] is a measure of the distance between two random distributions, which is equivalent to the difference of information entropy between two distributions. Inspired by this, we introduce the idea of relative entropy into image retrieval.

**Figure 2:**
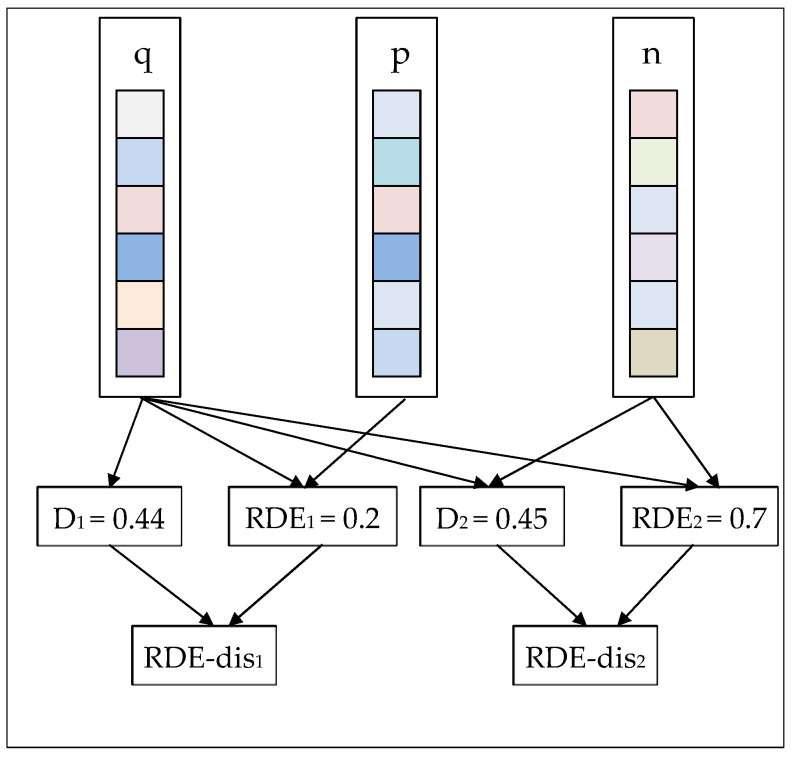
The core idea of relative distribution entropy (RDE)-loss. D represents the Euclidean distance between two image descriptors. RDE represents the relative distribution entropy between two image descriptors. RDE-distance is a new metric that combines Euclidean distance with relative distributed entropy. We called it the relative distribution entropy weighted distance, which can enhance the discrimination of image descriptors.

To solve the key problem mentioned above, we propose relative distribution entropy (RDE) to describe the distribution attributes of image descriptors. We combine the relative distribution entropy with the Euclidean distance [29] to build the relative distribution entropy weighted distance (RDE-distance). We fuse the RDE-distance into the contrastive loss and triplet loss to obtain the relative distributed entropy contrastive loss and the relative distributed entropy triplet loss. We call them the relative distributed entropy loss functions. To be more specific, we make three contributions, as follows:

Firstly, we propose a loss function modified by relative distribution entropy, called the relative distribution entropy loss function. Furthermore, the relative distribution entropy loss function contains the following two aspects: (1) Euclidean distance between the descriptors. (2) The differences in internal distribution state between descriptors. The core idea of the algorithm is illustrated in Figure 2. We combine the Euclidean distance with the relative distribution entropy to obtain the relative distribution entropy weighted distance (RDE-distance), which increases the discrimination between the image pair. We replace the Euclidean distance in the original contrastive loss and triplet loss with the relative distribution entropy weighted distance (RDE-distance) to obtain the relative distribution entropy loss function. 

Secondly, during the experiment, we use GeM pooling [30] and whitening post-processing [31].

Thirdly, we employ the fine-tuned network [30] to perform our experiments on different datasets to verify the effectiveness of our proposed method.

The organization of this work is as follows. Section 2 introduces the related work. Section 3 mainly introduces our proposed relative distribution entropy loss function. Specific experimental results and analyses will be presented in Section 4. Section 5 is a summary of the contributions and methods of this paper.

## 2. Related Work

### 2.1. Deep Metric Learning

Deep metric learning (DML) has become one of the most interesting research areas in machine learning. Metric learning aims to learn an embedding space. In this space, images are converted into embedding vectors. The distance between embedding vectors of positive samples is small, and the distance between embedding vectors of negative samples is large [11,12,13]. Now, deep metric learning has played a vital role in many areas, such as face recognition [32], pedestrian recognition [33], fine-grained retrieval [34], image retrieval [35], target tracking [36], and multimedia retrieval [37]. We will summarize the recent emergence of metric learning methods in the next section. Here, we introduce deep metric learning into image retrieval. Deep metric learning aims to learn a discriminative feature embedding f(x) for input image x. In other words, f(x) is the descriptor of the image. Formally, we define the Euclidean distance between two descriptors as $D_{x_{i},x_{j}}=\|f\left(x_{i}\right)-f\left(x_{j}\right)\;{\|}_{2}$.

#### 2.1.1. Contrastive Loss

The siamese network [14] is a typical pair-based method. Its embedding is obtained through contrastive loss. The purpose of contrastive loss is to reduce the Euclidean distance between positive samples and increase the Euclidean distance between negative samples. The equation of the contrastive loss function is illustrated in (1).

(1) $$L\left(x_{i},x_{j}\right)=\gamma D_{x_{i},x_{j}}{}^{2}+\left(1-\gamma \right)max{\left(0,m-D_{x_{i},x_{j}}\right)}^{2}$$

γ=1 if the sample $x_{j}$ is a positive sample, and γ=0 if the sample $x_{j}$ is a negative sample. m is the margin. This keeps negative distances above a certain threshold. 

#### 2.1.2. Triplet Loss

However, the above models only focus on the similarity of intra-class of the samples. To solve this problem, the triplet loss function [15] is proposed. Each triplet comprises a positive sample and a negative sample sharing the query. Triplet loss aims to learn an embedding space. In this space, the distance between the query and the negative sample is greater than the distance between the query and the positive sample. The triplet loss function is illustrated in (2).

(2) $$L\left(x,x^{+},x^{-}\right)=max\left(0,D_{x,x^{+}}{}^{2}-D_{x,x^{-}}{}^{2}+m\right)$$

Here, $D_{x,x^{+}}=\|f\left(x\right)-f\left(x^{+}\right){\|}_{2}$ represents the Euclidean distance between the descriptors of the positive sample and the query. Similarly, $D_{x,x^{-}}=\|f\left(x\right)-f\left(x^{-}\right){\|}_{2}$ represents the Euclidean distance between the descriptors of the negative sample and the query. m is the violate margin that requires that the negative distances to be larger than the positive distances.

#### 2.1.3. N-pair Loss

Triplet loss function only compares one negative sample and ignores the negative samples of other classes during the learning stage. As a result, the embedding vector of the query can only be promoted to maintain a large distance between the selected negative samples, but it cannot guarantee to maintain a large distance between the embedding vector and other non-selected negative samples.

N-pair loss [18] has improved the above problems. Unlike triplet loss function, N-pair loss considers the relationship between query samples and other negative samples of different classes within a mini-batch. The equation of the N-pair loss function is illustrated in (3).

(3) $$L\left(x,x^{+},{\left\{x\right\}}_{i=1}^{N-1}\right)=log\left(1+\sum_{i=1}^{N-1}exp\left(f{\left(x\right)}^{T}f\left(x_{i}\right)-f{\left(x\right)}^{T}f\left(x^{+}\right)\right)\right)$$

Each training tuple of N-pair loss is composed of N+1 samples: $\left\{x,x^{+},x_{1},x_{2},\ldots x_{N-1}\right\}$. $x^{+}$ is the positive sample. ${\left\{x\right\}}_{i=1}^{N-1}$ are the negative samples. 

#### 2.1.4. Lifted Structured Loss

The existing triplet loss methods cannot take full advantage of mini-batch SGD training. Lifted structured loss calculates loss [17] based on all positive and negative sample pairs in the training set (mini-batch). The lifted structured loss function is illustrated in Equation (4).

(4) $$L=\sum max\left(0,log\left(\sum_{\left(x_{i},x_{j}\right)\epsilon P}exp\left\{m-D_{x_{i},x_{j}}\right\}\right)+log\left(\sum_{\left(x_{i},x_{j}\right)\epsilon N}exp\left\{D_{x_{i},x_{j}}\right\}\right)\right)$$

where P is the set of positive samples in the training set, and N is the set of positive samples in the training set. m is the violate margin.

Although these loss functions calculate the distance of descriptors between images, they neglect the difference in internal distribution between the image pair. In this work, we propose a new loss function, which is called the relative distribution entropy loss function. We use the relative distribution entropy to reflect the difference in the descriptor distribution, and add this difference to the loss function. In this way, the new loss function can combine the Euclidean distance with the internal relative difference in the distribution state between the descriptors of the image pair. 

### 2.2. Application of Spatial Information in Image Retrieval

The performance of image retrieval has been greatly improved in recent years through the use of deep feature representations. However, most existing methods aim to retrieve images that are visually similar or semantically relevant to the query, without considering the spatial information. Before that, some researchers attempted to add spatial information into image retrieval algorithms to improve retrieval performance. Mehmood [38] proposed adding a local region and a global histogram to the BoW algorithm and combining them as the final descriptor. Krapac [39] used the BoW descriptor to encode the spatial part of the image, which improved the performance of the image classification. Koniusz [40] used spatial coordinate coding to represent and simplify the spatial pyramid to provide more compact image features. Sanchezet [41] added the spatial position information of features into the descriptor, which overcame the change in the proportion of retrieved objects and the change in the local area of the image. Liu [42] introduced the concept of spatial distribution entropy and combined it with the VLAD algorithm. These methods have fully introduced the spatial information into the image retrieval algorithm, and great effects are obtained. However, these methods all aimed at the improvement of traditional image descriptors. In this work, we attempt to add spatial information to the descriptor by optimizing the loss function of a deep convolutional network. 

### 2.3. Pooling and Normalization

The feature map generated by the deep retrieval framework reflects the color, texture, shape, and other characteristics of the image. Since the convolutional neural network needs to be integrated into a multi-dimensional feature descriptor before retrieval, the feature map of the convolutional layer needs to be further processed. It is required that the processed result retains the main features of the image while reducing the parameters and calculations of the next layer. Babenko [43] proposed the mean-pooling method, which sums the pixel values of the feature map to obtain an *N*-dimensional feature vector. Razavian [44] used max-pooling or mean-pooling on the feature descriptors and the result reduced MAC [44] descriptors in dimension with a series of normalization and PCA [45] whitening operations. Finally, the region feature vectors are summed to obtain a single image representation. In this work, we used the generalized mean-pooling [30]. We used Z to represent the input to the pooling layer and h to represent the pooling layer output. The mentioned pooling can be expressed as follows:

Max pooling (MAC vector):

(5) $$h^{\left(m\right)}={\left[h_{1}^{\left(m\right)}\ldots h_{k}^{\left(m\right)}\ldots h_{K}^{\left(m\right)}\right]}^{T},h_{k}^{\left(m\right)}=\underset{z\in Z_{k}}{max}\;z$$

Average pooling (SPoC vector [43]):

(6) $$h^{\left(a\right)}={\left[h_{1}^{\left(a\right)}\ldots h_{k}^{\left(a\right)}\ldots h_{K}^{\left(a\right)}\right]}^{T},h_{k}^{\left(a\right)}=\frac{1}{\left|Z_{k}\right|}\sum_{z\in Z_{k}}z$$

Generalized mean pooling (GeM [30]):

(7) $$h^{\left(g\right)}={\left[h_{1}^{\left(g\right)}\ldots h_{k}^{\left(g\right)}\ldots h_{K}^{\left(g\right)}\right]}^{T},h_{k}^{\left(g\right)}={\left(\frac{1}{\left|Z_{k}\right|}\sum_{z\in Z_{k}}z^{p_{k}}\right)}^{\frac{1}{p_{k}}}$$

where K is the number of feature maps, k means the channel of features and $\left|Z_{k}\right|$ is the number of feature values in the k-th channel feature map. The descriptor finally consists of a single value per feature map.$\;p_{k}$ is the pooling parameter, which can be manually set or learned. The superscript of $h_{k}^{\left(g\right)}\;$is the pooling method. m,a,g represent the max pooling, average pooling and generalized mean pooling, respectively. 

The max-pooling and mean-pooling are special cases of generalized mean-pooling. When$\;p_{k}=1$, it is the mean-pooling. On the contrary, when it is positive infinity, it is the max-pooling. Generalized mean-pooling with parameters can better adapt to the network and improve retrieval performance. 

In this work, we use $L_{2}$ normalization to balance the effect of the range of pixel values as follows:

(8) $$V_{L_{2}}=\frac{v_{i}}{\left\|v\right\|}$$

where v represents a vector, ‖v‖ represents the norm of the vector, and $v_{i}$ represents the value of the dimension on the vector.

### 2.4. Whitening

In large-scale image retrieval applications, high-dimensional global images typically require the use of PCA to reduce the dimensions of features for the next step. Jegou and Chum [45] studied the influence of PCA on the BoW and VLAD descriptor representations, and highlighted the use of multiple visual dictionaries for dimensionality reduction, thereby reducing the information loss of the dimensionality reduction process. In this work, whitening is used as a post-processing step. In this paper, the method of whitening is the linear discriminant projection proposed by Mikolajczyk and Matas [31].

The processing steps are divided into two parts. In the first part, the intra-class image feature vector is whitened. The whitening part is the reciprocal of the square root of the intra-class image pair (matched image pair) covariance matrix $C_{S}{}^{-\frac{1}{2}}$.

(9) $$C_{S}=\sum_{Y\left(x_{i},x_{j}\right)=1}\left(f\left(x_{i}\right)-f\left(x_{j}\right)\right){\left(f\left(x_{i}\right)-f\left(x_{j}\right)\right)}^{T}$$

where $f\left(x_{i}\right)$ and $f\left(x_{j}\right)$ are the descriptors of the image after pooling, $Y\left(x_{i},x_{j}\right)=1$ represents the image matched pair, and $C_{s}$ represents the covariance matrix of the matched image pair.

In the second part, the inter-class image feature is rotated. The rotating part is the eigenvector of the covariance matrix $C_{S}^{\frac{1}{2}}C_{D}C_{S}^{-\frac{1}{2}}$ between inter-class image pair (the non-matched image pair)

(10) $$C_{D}=\sum_{Y\left(x_{i},x_{j}\right)=0}\left(f\left(x_{i}\right)-f\left(x_{j}\right)\right){\left(f\left(x_{i}\right)-f\left(x_{j}\right)\right)}^{T}$$

where$\;f\left(x_{i}\right)$ and $f\left(x_{j}\right)$ are the descriptors of the image after pooling,$Y\left(x_{i},x_{j}\right)=0$ represents the non-matched image pair, and $C_{D}$ represents the covariance matrix of the non-matched image pair.

Then, we apply the projection $P=C_{S}{}^{-\frac{1}{2}}eig\left(C_{S}{}^{-\frac{1}{2}}C_{D}C_{S}{}^{-\frac{1}{2}}\right)$ to $P^{T}\left(f\left(x_{i}\right)-\mu \right)$, where μ is the mean GeM [30] vector to perform centering.

## 3. Method Overview

### 3.1. Calculation of Relative Distribution Entropy

We firstly introduce the concept of relative distribution entropy. Relative distribution entropy can better represent the distribution difference between two descriptors of image samples. The relative distribution entropy is derived from the relative entropy. Relative entropy can be computed as follows:

(11) $$R\left(P∥Q\right)=\sum P\left(x\right)log\frac{P\left(x\right)}{Q\left(x\right)}$$

P(x) and Q(x) are the two probability distributions on the random variable X. From this, we can get the equation of relative distribution entropy (RDE).

(12) $$P\left(f{\left(x_{i}\right)}^{m}\right)=\frac{histogram\left(f\left(x_{i}\right)\right)\left[m\right]}{dim}$$

(13) $$RDE\left(x_{i}∥x_{j}\right)=\sum_{m=1}^{n}\left(f{\left(x_{i}\right)}^{m}\right)log\frac{P\left(f{\left(x_{i}\right)}^{m}\right)}{Q\left(f{\left(x_{j}\right)}^{m}\right)}$$

where $x_{i},x_{j}$ represent the images. $f\left(x_{i}\right)$ represents the descriptor of image $x_{i}$. It is a normalized vector. $RDE\left(x_{i}∥x_{j}\right)$ is the relative distribution entropy of two images. In this work, we use histograms to describe the distribution of image descriptors. n is the number of bins, and is an adjustable parameter. $P\left(f{\left(x_{i}\right)}^{m}\right)\;$is the probability distribution in the $m^{th}$ bin. The equation for $Q\left(f{\left(x_{j}\right)}^{m}\right)$ is the same as for$\;P\left(f{\left(x_{i}\right)}^{m}\right)$. dim is the dimension of descriptors.

### 3.2. Relative Distribution Entropy Loss Function

From the above, we introduce the calculation of relative distributed entropy. Next, we show how to add the relative distribution entropy into the loss function. We add the relative distribution entropy to contrastive loss [14] and triplet loss [15] to build relative distribution entropy loss functions. 

Firstly, we introduce the fusion process of contrastive loss and relative distribution entropy. The equation of the contrastive loss function is shown in (14).

(14) $$L\left(x_{i},x_{j}\right)=\gamma D_{W}{\left(x_{i},x_{j}\right)}^{2}+\left(1-\gamma \right)max{\left(0,m-D_{W}\left(x_{i},x_{j}\right)\right)}^{2}$$

(15) $$D_{W}\left(x_{i},x_{j}\right)=\|f\left(x_{i}\right)-f\left(x_{j}\right)2_{\|}$$

where $D_{W}\left(x_{i},x_{j}\right)$ represents the Euclidean distance between the descriptors of the query $x_{i}$ and the sample $x_{j}$. γ=1 if the sample $x_{j}$ is a positive sample, and γ=0 if the sample $x_{j}$ is a negative sample. m is the margin.

Then, we add the relative distribution entropy to $D_{W}$ to get the $D_{NW}$:

(16) $$D_{NW}\left(x_{i},x_{j}\right)=\|f\left(x_{i}\right)-f\left(x_{j}\right){\|}_{2}+\alpha RDE\left(x_{i}∥x_{j}\right)$$

where α is the weighting parameter of the relative distribution entropy. As illustrated in Equation (16), we get the new distance metric. We call it the relative distribution entropy weighted distance (RDE-distance). We substitute $D_{NW}$ into (14) to get the new contrastive loss function, as is shown in Equation (17):

(17) $$L\left(x_{i},x_{j}\right)=\gamma D_{NW}{\left(x_{i},x_{j}\right)}^{2}+\left(1-\gamma \right)max{\left(0,m-D_{NW}\left(x_{i},x_{j}\right)\right)}^{2}$$

Similarly, we introduce the fusion process of triplet loss and relative distribution entropy. The equation of the contrastive loss function is shown in (18).

(18) $$L\left(x,x^{+},x^{-}\right)=max\left(0,D_{P}{\left(x,x^{+}\right)}^{2}-D_{N}{\left(x,x^{-}\right)}^{2}+m\right)$$

$D_{P}\left(x,x^{+}\right)$ is the Euclidean distance between the descriptors of the positive sample and the query. Similarly, $D_{N}\left(x,x^{-}\right)$ is the Euclidean distance between the descriptors of the negative sample and the query.

Then, we add the relative distribution entropy to $D_{P},D_{N}$ to get the$\;D_{NP},\;D_{NN}$.

(19) $$D_{NP}\left(x,x^{+}\right)=\|f\left(x\right)-f\left(x^{+}\right){\|}_{2}+\alpha RDE\left(x∥x^{+}\right)$$

(20) $$D_{NN}\left(x,x^{-}\right)=\|f\left(x\right)-f\left(x^{-}\right){\|}_{2}+\alpha RDE\left(x∥x^{-}\right)$$

We substitute the new Euclidean distances into Equation (18) and get the new triplet loss.

(21) $$L\left(x,x^{+},x^{-}\right)=max\left(0,D_{NP}{\left(x,x^{+}\right)}^{2}-D_{NN}{\left(x,x^{-}\right)}^{2}+m\right)$$

### 3.3. Network Architecture for Relative Distribution Entropy Loss Function

#### 3.3.1. CNN Network Architecture

We construct a CNN neural network to obtain the descriptor of the image. We only use the convolutional layers, discarding the fully connected layer. Convolution layers can extract features of images. The feature map obtained by the convolution layer is vectorized by the GeM pooling operation [30]. If the whitening operation is performed, the whitening is processed following the pooling layer. Whitening can reduce the correlation between features, and it can make the features share the same variance (covariance matrix is 1), which can greatly improve the image retrieval performance. Here, we use the $L_{w}\;$whitening [31] method. The last step is the normalization operation. The purpose of normalization is to make the preprocessed data limited to a certain range (such as [0,1] or [−1,1]), thereby eliminating the adverse effects caused by the singular sample data. The network architecture is pre-trained in ImageNet [46] network architecture.

Furthermore, we adopt network architectures such as ResNet [47] and AlexNet [9], and these two networks are also pre-trained on ImageNet [46].

**Figure 3:**
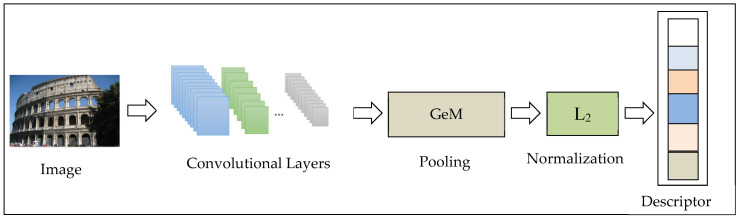
Convolutional neural network (CNN) network architecture.

The CNN network architecture is shown in Figure 3.

#### 3.3.2. Architecture of Training

**Figure 4:**
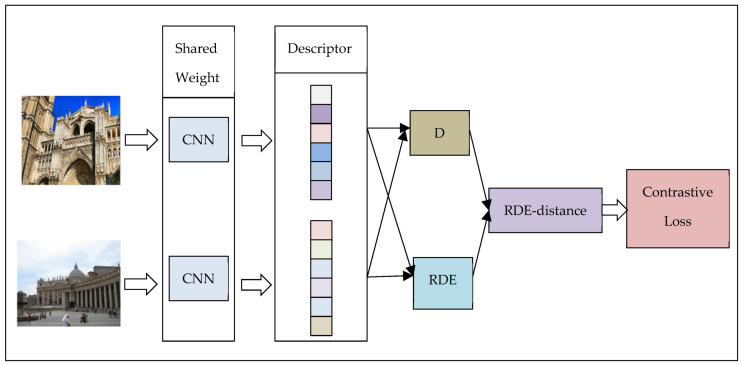
Training process using relative distribution entropy contrastive loss.

**Figure 5:**
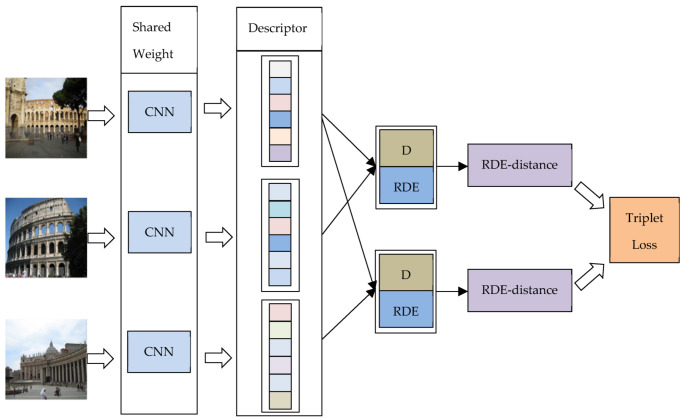
Training process using relative distribution entropy triplet loss.

The training procedure consists of multiple networks sharing the same weight. The architecture of CNN is introduced in the previous part. We added our newly proposed relative distribution entropy to the previous loss function, as shown in Figure 4 and Figure 5:

In Figure 4 and Figure 5, D represents the Euclidean distance between the two descriptors, and RDE represents the relative distribution entropy between the two descriptors. RDE-distance is the relative distribution entropy weighted distance obtained after the fusion of D and RDE.

## 4. Experiments and Evaluation

In this section, we discuss the implementation details of training and testing. Also, we analyze the experimental results and compare them with previous work.

### 4.1. Training Datasets

In this work, experimental training data are distilled from Retrieval-SFM-120K [48], which contains 7.4 million images. After clustering [49], we get about 20,000 images as the query seed. The structure-from-motion (SfM) algorithm constructs 1474 3D models from the training datasets. We removed the duplications and retained 713 of them, which contained more than 163,000 different images.

There are 91,642 training images in the dataset, and 98 cluster images that are the same or almost the same as the test dataset. Through the minimum hash and spatial verification methods mentioned in the clustering process, about 20,000 images are selected as query images, 18,1697 pairs of positive images and 551 training clusters, including more than 163,000 clusters [50] from the original dataset. The dataset contains all images from the Oxford 5k [51] and Paris 6k [52] datasets.

### 4.2. Training Configurations

In the experiments, we use the Pytorch deep learning framework to train the deep network model. We use ResNet [47], VGG [53] and AlexNet [9], which are all pre-trained on ImageNet [46].

In the experiment of relative distribution entropy contrastive loss, ResNet [47] and VGG [53] are trained using Adam learning strategy [54], while AlexNet [9] is trained using SGD. Our initial learning rate for Adam is $10^{-6}$, and the margin for ResNet and VGG are 0.95 and 0.9. We use an initial learning rate equal to $10^{-3}$ for SGD, and the margin for AlexNet is 0.75.

In the experiment of triplet loss, we also use ResNet [47], VGG [53], and AlexNet [9] to initialize the network. They are trained using the Adam learning strategy [54]. Our initial learning rate for Adam is $10^{-6}$. The margin for ResNet and VGG are 0.5, and the margin for AlexNet is 0.3. The size of the training image is not more than 362 * 362 while maintaining the aspect ratio of the original image. 

The experimental environment is an intel(R) i7-8700 processor, GPU with 12GB of memory, NVIDIA(R) 2080Ti graphics card, driver version 419.**. Operating system is Ubuntu 18.04 LTS, PyTorch version v1.0.0, CUDA version 10.0, CUDNN version 7.5. The time spent in each training cycle trained on our method on VGG, ResNet, and AlexNet is 0.48, 0.72, and 0.22 hours, respectively. During the testing phase, testing VGG, ResNet, and AlexNet networks takes 620, 990, and 277 seconds, respectively. There are subtle differences between different test sets. With the same computing power, the training time of our method is almost the same as that of other methods [30].

### 4.3. Datasets and Evaluation of Image Retrieval

We conduct our testing experiments on the following benchmark datasets frequently. Herein, we give the details of these datasets. 

Oxford5k [51] is a widely used landmark dataset consisting of 5062 building images from the Flickr dataset. It contains 11 famous landmarks in the Oxford area, and each landmark building has 55 query images.

Paris6k [52] contains 6392 images and is also one of the widely used datasets in the field of image retrieval. It collects many landmark buildings in Paris, and most of these images are from tourists. Similar to the Oxford 5k dataset, it also has 55 query images.

In addition, we use 100k interference images to fuse with the Oxford5k and Paris6k datasets to obtain Oxford105k [51] and Paris106k [52]. 

In the experiments, we use mean average precision (mAP) to measure the performance of image retrieval. 

### 4.4. Results and Analysis

#### 4.4.1. The Adjustment Process of Hyperparameter

In this experiment, two hyperparameters α and β are adjusted to obtain best performance. α is the weight of the relative distribution entropy. As mentioned in Section 3.2, when fusing the Euclidean distance with RDE, the weight α will affect the ratio of Euclidean distance and relative distribution entropy to the finally generated RDE-distance. As mentioned in Section 3.1, β is the total bin amount in the histogram during the calculation of RDE. β will affect the degree of differentiation of internal spatial distribution differences between two descriptors. However, a large β also increases the computational burden. The ability of RDE-distance to distinguish between two descriptors is determined by two factors α and β. The values of these two hyperparameters have a great impact on our experimental results. During our experiments, we adjust them to get the best performance. We use AlexNet and VGG networks for tuning and the GeM pooling [30]. The partially representative results are shown in Table 1.

Here, we take some representative results. In the relative distribution entropy contrastive loss, we take the value of α within 0.5–1, and the results show that the performance is the best when α=0.9. Additionally, we take the value of β within 10–100. When the value of β is large (β>50), the effect of adding relative distribution entropy is not obvious. After a large number of experiments, we can make the following conclusions. When α=0.90 and β=30, our performance achieve the best results on Oxford5k and Pairs6k. The performance achieves 88.00% and 88.12% on VGG. On AlexNet, the performance achieves 68.22% and 80.07%. Therefore, we set β to 30 and α to 0.9 in ResNet and VGG as our final hyperparameters. 

#### 4.4.2. Comparison of MAC, SPoC, and GeM

In this section, we combine the relative distribution entropy contrastive loss function with the current most advanced pooling methods, GeM [30], MAC [44] and SPoc [43], for end-to-end training. In this experiment, we use the AlexNet for training. The experimental results are shown in Table 2. 

The conclusions drawn from Table 2 are as follows. The results in the table indicate that the experiment results using GeM pooling [30] on AlexNet are superior to the other two pooling methods. We get the results of 60.79%, 68.22%, 75.29% and 80.07%, which are the maximum values on the different datasets. In the next experiments, we will use the GeM pooling [30] method for training.

#### 4.4.3. Comparison of Relative Distribution Entropy Triplet Loss and Triplet Loss

In this section, we present the experimental results of our method in triplet loss and compare them with the previous method [30]. We perform comparison tests on VGG and ResNet. Using the same pooling method, experimental steps, and network model, we compare the relative distribution entropy triplet loss with the traditional triplet loss. The comparison results are shown in Table 3.

From Table 3, the result indicates that when we experiment on VGG, our proposed method obtains the best performance on all these datasets, with 82.39%, 83.07%, 83.61%, and 85.45%. The same conclusion is obtained when we perform the experiments on ResNet. We get the results of 82.88%, 86.54%, 89.33%, and 91.97%, which is the best performance amongst the datasets. In this experience, we combine the relative entropy with the Euclidean distance into relative distributed entropy weighted distance, which is a new metric. We put this new metric into the triplet loss function, and experiments have proven that our method is greatly effective.

#### 4.4.4. Comparison with State-of-Art

In this section, we compare the relative distribution entropy contrastive loss with the latest methods. The performance comparison is shown in Table 4. The results of other methods are given by referring to the results in their papers. From Table 4, it can be learned that our proposed method attains better performance on multiple datasets. As shown in the table, we divide the existing networks into two categories: (1) using fine-tuning networks (yes) and (2) not using fine-tuning networks (no). When using the VGG network, compared with the RMAC [55], relative distribution entropy contrastive loss provides a significant improvement of +4.9% and +1.0% on the Oxford5k and Paris6k datasets, respectively. Compared to the latest release, our method also has performance improvements. When using ResNet, our experimental results achieve +0.6% growth compared to GeM [30] on Oxford 5k. Our method also shows superior performance on large-scale datasets. When using the VGG network, our experimental results achieved +0.2% growth compared to GeM [30] on Oxford 105k. When using the ResNet network, our experimental results achieve +0.3% growth compared to GeM [30] on Oxford105k. Our method shows more obvious performance improvements after adding re-ranking and query expansion. Under the VGG, the gain over GeM + αQE [30] is +0.1% and +0.5% on the Paris 6k dataset, respectively. Under the ResNet, the our method achieved mAP of 91.7%, 89.7%, 96.0%, and 92.1% and offered over 91.0%, 89.5%, 96.7%, and 91.9% gain over the GeM+αQE [37] on Oxford 5k, Oxford 105k, Paris 6k and Paris 106k datasets, respectively.

## 5. Conclusions

In this paper, we discuss the deficiency of traditional loss functions in spatial distribution differences. To make up for the lack of spatial distribution differences in the descriptors of image pair, the concept of relative distribution entropy (RDE) is presented. The calculation process of the relative distribution entropy is introduced in Section 3. Next, we combine Euclidean distance and relative distribution entropy to obtain a new similarity measurement, called relative distribution entropy weighted distance (RDE-distance). We combine RDE-distance with contrastive loss and triplet loss to obtain relative distribution entropy contrastive loss and relative distribution entropy triplet loss. We train the entire framework in an end-to-end manner, and the results of extensive experiments prove that our new method achieves the state-of-the-art performance. 

Our method mainly focuses on how to fuse Euclidean distance and spatial information. Here, we introduce relative distribution entropy to describe spatial information. We would like to focus on other fusion methods instead of the existing linear fusion. In addition, we would concentrate on adding our relative distribution entropy to other loss functions in future work.

## Figures and Tables

**Table 1:** Experimental results of hyperparameter comparison in the relative distribution entropy contrastive loss function. The best results would be highlighted in bold.

Network	α	β	Oxford5k	Oxford5k(W)	Pairs6k	Pairs6k(W)
AlexNet	0.50	10	58.10	67.60	71.64	79.60
	0.75	20	60.87	67.19	75.33	79.43
	0.85	25	60.79	67.93	**75.60**	79.59
	**0.90**	**30**	**61.32**	**68.22**	75.29	**80.07**
	1.00	50	60.27	67.72	74.88	80.10
VGG	0.85	30	84.62	87.83	82.40	88.01
	0.85	100	76.18	83.04	81.71	87.11
	**0.90**	**30**	**85.09**	**88.00**	**82.69**	**88.12**

**Table 2:** Comparative results of different pooling methods on AlexNet. The best results would be highlighted in bold.

Net	Oxford5k	Oxford5k(W)	Pairs6k	Pairs6k(W)
SPoC [43]	41.83	55.34	55.49	68.61
MAC [44]	47.50	55.95	62.16	71.30
GeM [30]	**60.79**	**68.22**	**75.29**	**80.07**

**Table 3:** Performance comparison of relative distribution entropy triplet loss and triplet loss. The best results would be highlighted in bold.

Loss	Network	Oxford5k	Oxford5k(W)	Pairs6k	Pairs6k(W)
Triplet loss [30]	VGG	81.48	82.80	82.79	84.78
Ours		**82.39**	**83.07**	**83.61**	**85.45**
Triplet loss [30]	ResNet	81.49	85.33	87.70	91.11
Ours		**82.88**	**86.54**	**89.33**	**91.97**

**Table 4:** Comparison of our method with the state-of-art image retrieval methods. The best results would be highlighted in bold.

Net	Method	F-tuned	Oxford5k	Oxford105k	Pairs6k	Pairs106k
VGG	MAC [44]	no	56.4	47.8	72.3	58.0
	SPoC [43]	no	68.1	61.1	78.2	68.4
	Crow [56]	no	70.8	65.3	79.7	72.2
	R-MAC [52]	no	66.9	61.6	83.0	75.7
	BoW-CNN [57]	yes	73.9	59.3	82.0	64.8
	NetVLAD [58]	yes	71.6	-	79.7	-
	Fisher [59]	yes	81.5	76.6	82.4	-
	R-MAC [55]	yes	83.1	78.6	87.1	79.7
	GeM [30]	yes	87.9	83.3	87.7	**81.3**
	ours	yes	**88.0**	**83.5**	**88.1**	79.9
Res	R-MAC [52]	no	69.4	63.7	85.2	77.8
	GeM [30]	yes	87.8	84.6	92.7	**86.9**
	ours	yes	**88.4**	**84.9**	**92.7**	86.3
Re-ranking(R) and Query Expansion(QE)						
VGG	Crow + QE [56]	no	74.9	70.6	84.8	79.4
	R-MAC+R+QE [52]	no	77.3	73.2	86.5	79.8
	BoW-CNN+R+QE [57]	no	78.8	65.1	84.8	64.1
	R-MAC+QE [55]	yes	89.1	87.3	91.2	86.8
	GeM+αQE [30]	yes	91.9	89.6	91.9	**87.6**
	ours	yes	**92.0**	**89.6**	**92.4**	87.3
Res	R-MAC+QE [52]	no	78.9	75.5	89.7	85.3
	R-MAC+QE [60]	yes	90.6	89.4	96.0	93.2
	GeM+QE [30]	yes	91.0	89.5	95.5	91.9
	ours	yes	**91.7**	**89.7**	**96.0**	**92.1**
